# Differential role of MAX2 and strigolactones in pathogen, ozone, and stomatal responses

**DOI:** 10.1002/pld3.206

**Published:** 2020-02-28

**Authors:** Maria Kalliola, Liina Jakobson, Pär Davidsson, Ville Pennanen, Cezary Waszczak, Dmitry Yarmolinsky, Olena Zamora, E. Tapio Palva, Tarja Kariola, Hannes Kollist, Mikael Brosché

**Affiliations:** ^1^ Faculty of Biological and Environmental Sciences Viikki Plant Science Centre University of Helsinki Helsinki Finland; ^2^ Institute of Technology University of Tartu Tartu Estonia; ^3^ Organismal and Evolutionary Biology Research Programme Faculty of Biological and Environmental Sciences Viikki Plant Science Centre University of Helsinki Helsinki Finland; ^4^ LUMA Centre Päijät‐Häme University of Helsinki Lahti Finland

**Keywords:** abscisic acid, CO_2_ signaling, guard cell signaling, MAX2, pathogen defense, Strigolactone

## Abstract

Strigolactones are a group of phytohormones that control developmental processes including shoot branching and various plant–environment interactions in plants. We previously showed that the strigolactone perception mutant *more axillary branches 2 (max2)* has increased susceptibility to plant pathogenic bacteria. Here we show that both strigolactone biosynthesis (*max3* and *max4*) and perception mutants (*max2* and *dwarf14*) are significantly more sensitive to *Pseudomonas syringae* DC3000. Moreover, in response to *P. syringae* infection, high levels of SA accumulated in *max2* and this mutant was ozone sensitive. Further analysis of gene expression revealed no major role for strigolactone in regulation of defense gene expression. In contrast, guard cell function was clearly impaired in *max2* and depending on the assay used, also in *max3*, *max4,* and *d14* mutants. We analyzed stomatal responses to stimuli that cause stomatal closure. While the response to abscisic acid (ABA) was not impaired in any of the mutants, the response to darkness and high CO_2_ was impaired in *max2* and *d14‐1* mutants, and to CO_2_ also in strigolactone synthesis (*max3*, *max4*) mutants. To position the role of MAX2 in the guard cell signaling network, *max2* was crossed with mutants defective in ABA biosynthesis or signaling. This revealed that MAX2 acts in a signaling pathway that functions in parallel to the guard cell ABA signaling pathway. We propose that the impaired defense responses of *max2* are related to higher stomatal conductance that allows increased entry of bacteria or air pollutants like ozone. Furthermore, as MAX2 appears to act in a specific branch of guard cell signaling (related to CO_2_ signaling), this protein could be one of the components that allow guard cells to distinguish between different environmental conditions.

## 1 INTRODUCTION

Strigolactones are best known for their role in regulation of shoot branching by influencing polar auxin transport (Crawford et al., 2010; Hayward, Stirnberg, Beveridge, & Leyser, 2009; Waters, Makarevitch, Noshay, Burghardt, & Hirsch, 2017). Strigolactones also affect root development (Al‐Babili & Bouwmeester 2015, Waldie, McCulloch, & Leyser, 2014; Waters et al., 2012). Multiple plant–environment interactions are influenced by strigolactones. Parasitic weeds (Striga spp) recognize strigolactones secreted from plant roots, which stimulates their germination (Yoneyama et al., 2015). Strigolactones also regulate senescence and responses to salinity and light stress (Gomez‐Roldan et al., 2008; Shen, Luong, & Huq, 2007; Umehara et al., 2008). Moreover, recently Stes et al., (2015) demonstrated that strigolactones contribute to tolerance to the leafy gall syndrome (caused by *Rhodococcus fascians*), which indicates their role in regulating plant–pathogen interactions. Strigolactones also alter drought tolerance by affecting stomatal conductance (Bu et al., 2014; Ha et al., 2014).

Strigolactones are synthesized mainly in roots and transported to shoots; however, the route of transport is not clear (Kohlen et al., 2011; Xie et al., 2015). Both strigolactone synthesis and perception involve *MORE AXILLARY GROWTH (MAX)* proteins that act in a single pathway. MAX1, MAX3, and MAX4 catalyze strigolactone biosynthesis, while the perception requires MAX2 and DWARF14 (D14)—the receptor of strigolactone (Al‐Babili & Bouwmeester, 2015; Chevalier et al., 2014; Waters et al., 2012). MAX2 is an F‐box protein that targets proteins for destruction as part of the ubiquitin–proteasome system (Lechner, Achard, Vansiri, Potuschak, & Genschik, 2006; Stirnberg, Sande, & Leyser, 2002). D14 represents a novel system for hormone perception, as this protein both acts as a receptor for strigolactone and degrades strigolactone (Seto et al., 2019). Binding of strigolactone to D14 facilitates the interaction of D14 with SCF^MAX2^ (SKP1‐CUL1‐F‐box), an E3 ligase functioning in ubiquitination (Lv et al., 2018). Targets for MAX2‐directed protein degradation include SUPPRESSOR OF MORE AXILLARY GROWTH2‐LIKE6 (SMXL6), SMXL7, and SMXL8 (Wang et al., 2015). As D14 also degrades strigolactone, this represents a direct way for terminating the strigolactone signal (Seto et al., 2019).

Stomata play a central role in carbon assimilation and stress responses as they regulate the uptake of CO_2_ which is inevitably connected to the evaporative loss of water. Moreover, open stomata provide an entry point for air pollutants and plant pathogens (Melotto, Zhang, Oblessuc, & He, 2017; Vainonen & Kangasjärvi, 2015). Guard cells which form the stomatal pore respond to various endogenous and environmental stimuli by regulating their volume that in turn has a direct impact on the aperture of stomatal pores. Stomatal closure is induced by abscisic acid (ABA), pathogen‐associated molecular patterns (PAMPs), high carbon dioxide (CO_2_) concentration, darkness, drop in relative air humidity and air pollutants such as ozone (Melotto, Underwood, Koczan, Nomura, & He, 2006; Merilo et al., 2013). ABA biosynthesis starts from carotenoids, and ABA2 (ABSCISIC ACID DEFICIENT2) catalyzes the conversion of xanthoxin to abscisic aldehyde (González‐Guzmán et al., 2002). Analysis of the *aba2* mutant that express *ABA2* with either guard cell‐ or phloem‐specific promoter shows that both promoters could restore ABA levels and functional ABA responses, demonstrating effective transport of ABA between tissues (Merilo et al., 2018). ABA‐induced stomatal closure is initiated after binding of the hormone by PYR/PYL/RCAR receptors leading to inactivation of PP2C phosphates and followed by release of SNF‐related protein kinases (SnRK2s) such as OST1 (OPEN STOMATA1). OST1 together with Ca^2+^‐dependent protein kinases activate SLAC1 (SLOW ANION CHANNEL1) leading to stomatal closure (Merilo et al., 2013). Another protein, GUARD CELL HYDROGEN PEROXIDE‐RESISTANT1 (GHR1), is required for stomatal closure and is proposed to act as a scaffold bringing together various proteins needed to initiate stomatal closure (Hua et al., 2012; Sierla et al., 2018).

We previously showed that the strigolactone perception mutant *max2* has increased susceptibility to plant pathogenic bacteria (*Pseudomonas syringae*) as a result from more open stomata and impaired stomatal closure in response to infection (Piisilä et al., 2015). Further, we demonstrated that the *max2* mutant also exhibits other stress‐related phenotypes such as decreased tolerance to apoplastic reactive oxygen species (ROS), changes in stress‐related gene expression, and hormonal signaling, that is, increased salicylic acid levels (Piisilä et al., 2015). However, as MAX2 is known to participate in several signaling pathways and acts as a central regulator in both strigolactone and karrikin signaling (Li et al., 2017), we set out to clarify the role of strigolactones in plant defense responses by analysis of strigolactone biosynthesis mutants (*max3, max4*) and their receptor (D14). To this end, we analyzed the role of the strigolactone pathway in pathogen sensitivity, defense to ROS and stomatal regulation using single and double mutants defective in various steps of strigolactone biosynthesis and perception. Furthermore, the possible interaction between ABA and strigolactone signaling was assessed with a new set of double mutants.

## 2 MATERIALS AND METHODS

### 2.1 Plant material

All mutants used in this study were in the Col‐0 genetic background. The following mutants were obtained from the Nottingham Arabidopsis Stock Centre: *max2‐1* (Stirnberg et al., 2002), *max2‐4* (SALK_028336), *max3‐9* (Booker et al., 2004), *max3‐11* (SALK_023975), *max4‐1* (Sorefan et al., 2003), and *max4‐7* (SALK_082552), *ost1‐3* (*srk2e*, SALK_008068; Yoshida et al., 2002), *aba2‐11* (González‐Guzmán et al., 2002), *d14‐1* (CS913109; Waters et al., 2012), *sid2‐2* (Wildermuth, Dewdney, Wu, & Ausubel, 2001). The *d14‐seto5* and *max2‐1 d14‐seto5* (Chevalier et al., 2014) were obtained from Pilar Cubas. The *ghr1‐3* (GK_760C07; Sierla et al., 2018) was donated by Jaakko Kangasjärvi. All mutants were genotyped by PCR‐based markers (Table S1).

The double mutants *aba2‐11 max2‐4*, *ghr1‐3 max2‐4,* and *ost1‐3 max2‐4* were generated by crossing the respective single mutants with *max2‐4* (pollen donor). Double homozygous plants were identified in F2 segregating progenies by PCR with gene‐specific primers (Table S1).

### 2.2 Growth conditions

Growth conditions in University of Tartu (the gas exchange experiments). Arabidopsis seeds were planted in a soil pot covered by a glass plate with a hole through which the plants were grown as described by Kollist et al., (2007). The soil mixture contained 2:1 peat:vermiculite. Plants were grown in growth chambers in a 12‐hr photoperiod, 23°C/18°C temperature, 150 µmol m^−2^ s^−1^ light, and 70% relative humidity.

Growth conditions in University of Helsinki (all other experiments). Seeds were sown on a 1:1 peat:vermiculite mixture, vernalized in the dark for 2 days at 4°C, and germinated for 1 week. Next, plants were transferred to fresh pots to grow individually. Plants were grown in a growth room in 12‐hr light period (220 µmol m^−2^ s^−1^), 23°C/18°C day/night temperature, and 70% relative humidity. One to two weeks before the experiment, plants were moved into a growth chamber with similar temperature/light conditions.

For gene expression experiments with plants treated with GR24, seeds were surface sterilized with 70% ethanol and 2% Triton X‐100, rinsed 3 times with 99% ethanol, and dried on a filter paper. Plants were grown in vitro on ½ MS plates for 10 days in 16‐hr light/8‐hr dark cycle (110 µmol m^−2^ s^−1^), 23°C/18°C day/night temperature.

### 2.3 Stomatal aperture

Stomata were analyzed with a method by Chitrakar and Melotto (2010) in which the stomata are dyed with propidium iodide, and the visualized stomata were measured from the microscope images with ImageJ (see also Piisilä et al., 2015).

### 2.4 Pathogen assays

*Pseudomonas syringae* pv. *tomato* DC3000 were grown in King's B media at 28°C overnight, and the bacterial cells were collected by centrifugation at 6,000 rpm for 8 min and washed with 10 mM MgCl_2_. The centrifugation was repeated, and the bacteria were suspended in 10 mM MgCl_2_. OD_600_ was adjusted to 0.1, which equals to 10^7^ cfu/ml of bacteria in spray medium. To reduce surface tension, 0.02% Silwet/L77 was added just before inoculation. Next, 4‐ to 5‐week‐old plants were sprayed equally until their leaves were saturated with the spray medium. After inoculation, plants were covered with plastic to maintain the humidity. The amount of bacterial cells was determined at 1.5 hr and 48 hr postinoculation. For each biological replicate, three leaf disks from three separate leaves were analyzed. Leaf disks were surface sterilized with 70% ethanol, washed with MQ water, and ground in 0.2 ml of 10 mM MgCl_2_, after which the volume was adjusted to 1 ml. From the dilution series, the aliquots of different dilutions were pipetted to King's B media plates and grown for 2 days at 28°C.

### 2.5 Measurement of free SA levels

Free salicylic acid was measured using a modified biosensor‐based method based on a protocol described by DeFraia, Schmelz, and Mou (2008). The bacterial biosensor strain *Acinetobacter* sp. ADPWH*_lux* (Huang et al., 2006, 2005) was grown overnight in LB medium at 37°C, after which the culture was diluted 1:20 and grown to an OD_600_ of 0.4. Leaf samples of 30‐day‐old plants were collected 27 hr postspray inoculation with *Pst* DC3000 (OD_600_ = 0.2). A leaf disk (9 mm diameter) was cut from the 5th, 6th, and 7th leaf of each plant, and the three disks were homogenized in 200 µl of LB medium. To count bacterial cfu, we took 8 µl aliquot, and the rest of the sample was centrifuged for 15 min at 21,000 *g*. Next, 20 µl of the supernatant was mixed with 30 µl of LB medium and 50 µl of 1:100 diluted biosensor culture (OD_600_ = 0.004) on a white 96‐well plate. The plate was incubated at 37°C for one hour without shaking, after which the luminescence was measured with Perkin Elmer EnSpire 2300 plate reader. For standard curve, 0, 5, 10, 15, and 20 ng of sodium salicylate in 30 µl of LB medium were mixed with 20 µl of plant extract from *sid2‐2* plants and 50 µl of the diluted biosensor strain. The standard curve samples were measured at the same time as the samples. Due to non‐linearity of the standard curve values, separate linear best‐fit models were fitted for low (0–10 ng) and high (10–20 ng) amounts of salicylic acid standard as described by DeFraia et al., (2008). The luminescence values of the samples were converted to estimated masses of salicylic acid based on the standard curves and reported as ng/cm^2^. Statistical analysis was performed in R programming environment, and figures were prepared with ggplot2 package in R (R Core Team 2018, Wickham, 2016).

### 2.6 Ozone exposure and ion leakage analysis

The ozone exposure was conducted with 350 nl/L of ozone gas for 6 hr on 4‐ to 5‐week‐old plants as described by Overmyer et al., 2000. The relative ion leakage was measured as conductivity in 0, 6, and 24 hr after beginning of ozone exposure. There were four biological repeats for each time point from each plant line. The conductivity was measured using Horiba Twin Cond Conductivity Meter B‐173.

### 2.7 Expression analysis by qPCR

*Pseudomonas syringae* pv. *tomato* DC3000 bacteria were grown as described previously, and infection was done with OD_600_ = 0.1 on 4‐ to 5‐week‐old plants. The samples were taken 0 (i.e., non‐infected), 3, 6, 24, and 48 hr after the infection.

GR24 (Chiralix) was dissolved in DMSO, and a solution of 10 µM GR24 and 0.01% of Silwet‐L77 was poured into the wells of a 6‐well multi‐plate. The control solution consisted of the corresponding amounts of DMSO and Silwet‐L77. Whole intact 10‐day‐old in vitro grown Col‐0 seedlings were put into the wells and samples were collected after 3 hr.

RNA was isolated with GeneJET Plant RNA Purification Mini Kit (Thermo Scientific). RNA (3 µg in GR24 assay/2 μg in Pseudomonas infection) was DNAse‐treated and reverse‐transcribed with Maxima RT and Ribolock Rnase inhibitor (Thermo Scientific) according to the manufacturer instructions. After cDNA synthesis, the reactions were diluted to a final volume of 100 µl. qPCR was performed in triplicate using 5x HOT FIREPol EvaGreen qPCR Mix Plus (Solis Biodyne). The cycle conditions with Bio‐Rad CFX384 were as follows: 1 cycle initiating with 95°C 10 min, 50 cycles with 95°C 15 s, 60°C 30 s, 72°C 30 s, and ending with melting curve analysis. Normalization of the data was performed in qBase 3.0 (Biogazelle), with the reference genes PP2AA3, TIP41, and YLS8 in GR24 assay/ F‐box protein AT5G15710 in Pseudomonas infection. Primer amplification efficiencies were determined in qBase from a cDNA dilution series. All primers are listed in Table S1.

### 2.8 Stomatal conductance

The porometer measurements were done on 5‐ to 6‐week‐old plants with an AP4 porometer (Delta‐T Devices). The plants were measured according to the phenotype‐based growth stage (Boyes et al., 2001), and 2–3 leaves per each growth stage were measured from each plant, and altogether, a minimum 20 plants were measured per each plant line. The age of the plants was 5–6 weeks when the rosettes reached their maximal size but the flower buds were not yet visible.

The basal level of whole‐plant transpiration, stomatal conductance, and stomatal responses to CO_2_, darkness, and 5 µM ABA foliar spray were measured with a custom‐made gas exchange measurement device at the University of Tartu as described in details by Kollist et al., 2007. Transpiration reflects the amount of H_2_O (moles m^−2^ s^‐1^) that exits the plant, and stomatal conductance (mmol m^−2^ s^‐1^) reflects the resistance imposed by the stomata on gas flux between the intercellular airspaces and atmosphere, and is calculated as the ratio of the gas flux (CO_2_ or water vapor) to the concentration gradient of the gas between those two locations. The age of the plants was 3–4 weeks when they were measured. Due to the bushy phenotype of several studied mutants, the rosette area of all plants was calculated by separating the leaves and measuring individually. Prior to the experiment, plants were acclimated in the measurement cuvettes at ambient CO_2_ concentration (~400 ppm), 100 μmol m^−2^ s^−1^ light, and ambient humidity (RH 65%–80%) for at least 1 hr or until stomatal conductance was stable.

ABA‐induced stomatal closure was induced by foliar spray with 5 μM ABA solution (5 μM ABA, 0.012% Silwet‐L77, 0.05% ethanol). At time point T = 0 min, plants were removed from the measuring cuvettes and sprayed with either 5 μM ABA solution or control solution (0.012% Silwet‐L77, 0.05% ethanol). Thereafter, plants were returned to the cuvettes and stomatal conductance was monitored for 40 min.

Foliar spray with GR24 was conducted as follows: At time point T = 0 min, acclimated plants were removed from the measuring cuvette, and 10 μM GR24 solution (10 µM GR24, 0.012% Silwet‐L77 solution, 0.02% DMSO) was applied with a spray bottle 5 times approx. 30 cm from the rosette so that the plant would look slightly wet. Next, plants were returned to the measuring cuvettes to dry and starting from T = 8 min stomatal conductance was monitored for 56 min.

Statistical analyses of gas exchange data were performed with Statistica, version 7.1 (StatSoft Inc). All effects were considered significant at *p* < .05.

### 2.9 Accession numbers

ABA2 – AT1G52340, MAX2 – AT2G42620, OST1 – AT4G33950, GHR1 – AT4G20940, MAX3 – AT2G44990, MAX4 – AT4G32810, D14 – AT3G03990, ICS1/SID2 – AT1G74710, PR1 – AT2G14610, FRK1 – AT2G19190, GRX480 – AT1G28480, AXR3/IAA17 – AT1G04250, PP2AA3 – AT1G13320, YLS8 – AT5G08290, TIP41 – AT4G34270.

## 3 RESULTS

### 3.1 Strigolactone affects sensitivity to pathogens in Arabidopsis

Plant defense against pathogens involves a network of interacting signaling pathways where several plant hormones are key components (Overmyer, Vuorinen, & Brosche, 2018). Recently, strigolactones were identified as an additional component that regulates drought and pathogen responses (Bu et al., 2014; Ha et al., 2014; Piisilä et al., 2015). Here, we further explored the mechanism of strigolactones in sensitivity to pathogens by analysis of strigolactone receptor (D14) mutants and strigolactone synthesis (MAX3 and MAX4) mutants in order to find biological processes regulated by strigolactones.

**Figure 1:**
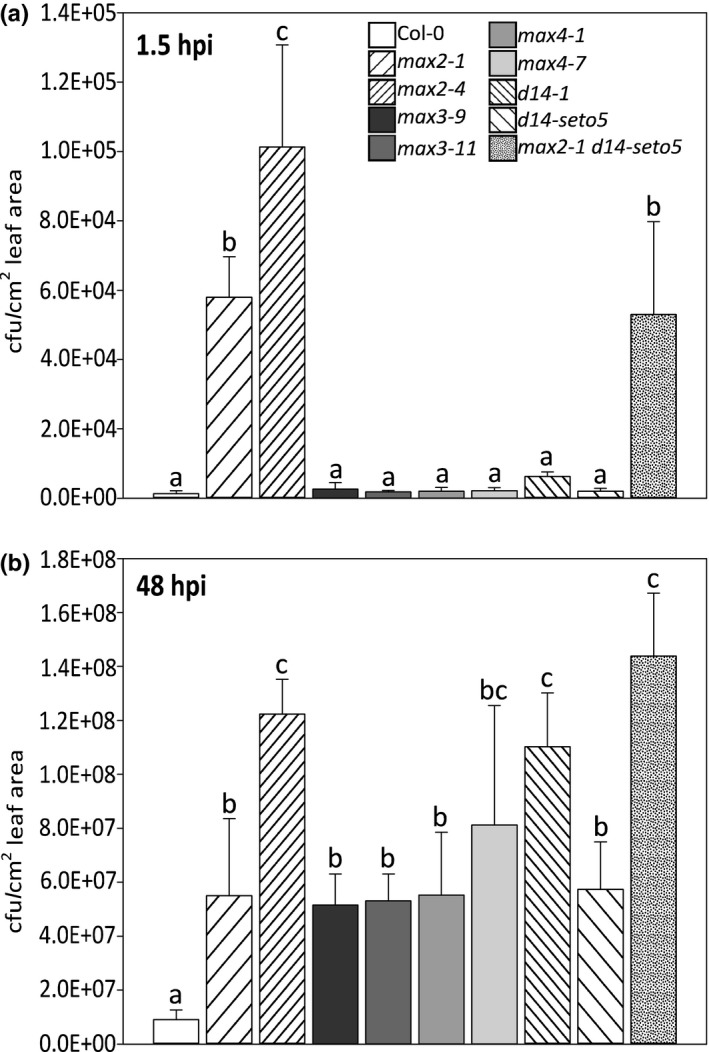
Bacterial calculation after infection with *Pseudomonas syringae* DC3000. The infection was done by spraying followed by calculation of bacteria at 1.5 hr and 48 hr postinoculation. In each experiment, four plants per line and three leaves per plant were used to measure the bacterial concentration. The experiment was repeated three times with similar results. The results are shown as means ± *SE*. In statistical analysis, we compared if Col‐0 significantly differs from mutants; first, a logarithmic transformation was conducted on the data and then univariate analysis of variance combined to Hochberg post hoc test

Spray infection with *P. syringae* DC3000 allows quantification of pathogen sensitivity in relation to stomatal immunity, as the bacteria need to enter the plant through stomata to multiply (Melotto et al., 2006). Therefore, we investigated the pathogen sensitivity of strigolactone synthesis and perception mutants. Interestingly, 1.5 hr postinoculation (hpi), only *max2* mutants exhibited increased sensitivity to *P. syringae* DC3000 infection (Figure 1; OD_600_ = 0.1) compared to Col‐0. However, at a later time point (48 hpi) strigolactone sensing (*max2*), the receptor (*d14*) and synthesis (*max3* and max*4*) mutants were all more sensitive to *P. syringae* DC3000 (Figure 1). Moreover, at 48 hpi some differences were observed between the mutants, notably, the *max2‐4* allele (a T‐DNA line) had a stronger phenotype than an EMS (ethyl methanesulfonate) mutant *max2‐1* (Stirnberg et al., 2002). Similarly, the T‐DNA knock‐out line *d14‐1* had a stronger phenotype than an EMS mutant *d14‐seto5* (Chevalier et al., 2014). In order to assess the role of stomatal openness in the infection, a similar experiment was also done in an inverted light rhythm, that is, the plants were infected in darkness when the stomata are normally closed, but the result was rather similar to the one obtained when infection was performed in normal light conditions (Figure S1). Therefore, we conclude that both strigolactone biosynthesis and perception mutants were all significantly more sensitive to *P. syringae* DC3000 spray infection than Col‐0 at 48 hpi.

### 3.2 *max2* mutants accumulate high level of free salicylic acid in response to *P. syringae* DC3000

**Figure 2:**
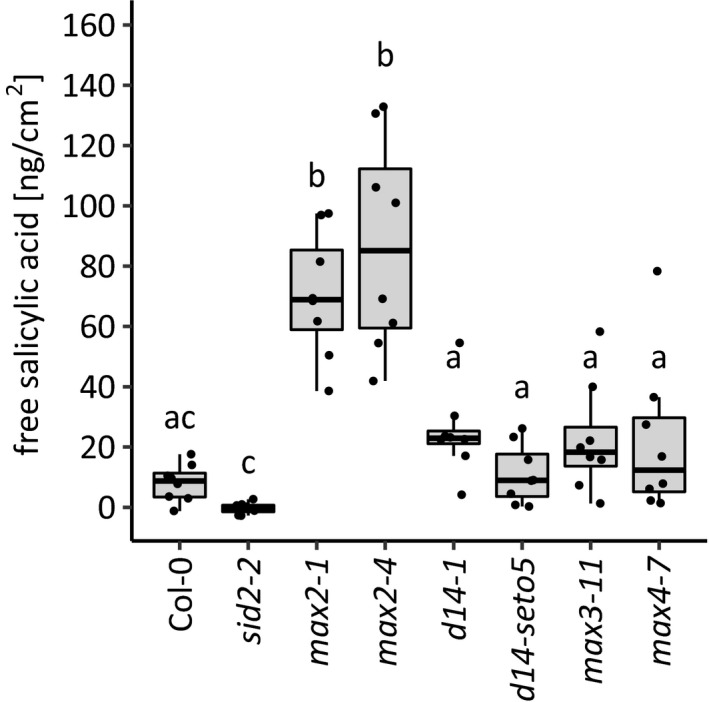
Accumulation of free salicylic acid in response to *Pseudomonas syringae* DC3000. Wild‐type (Col‐0), strigolactone signaling mutants (*max2‐1*, *max2‐4*, *d14‐1* and *d14‐seto5*) and strigolactone biosynthesis mutants (*max3* and *max4*) were infected with *P. syringae* (OD_600_ = 0.2) by spraying. SA was measured using a biosensor‐based method based on the protocol by DeFraia et al., (2008). Salicylic acid biosynthesis mutant (*sid2‐2*) was included as a control for accumulation of free salicylic acid. Salicylic acid accumulation was measured 27 hr postinoculation. Each dot represents one plant. In total, seven to eight plants were used per line. The experiment was performed three times with similar results. Box plots are summarizing data by showing the median, and first and third quartiles. Whiskers are extending to a maximum of 1.5 × interquartile range beyond the box. Different letters indicate significant differences (*p* < .05) as determined by Kruskal–Wallis rank sum test followed by pairwise Wilcoxon rank sum tests with multiple testing correction to p‐values using Holm method

The plant hormone salicylic acid (SA) has a key role in defense against pathogen infection (Fu & Dong, 2013, Pieterse et al., 2012). Therefore, we measured free SA levels in Col‐0 and the strigolactone biosynthesis/perception mutants at 27 hpi after spray infection with *P. syringae* DC3000 using OD_600_ = 0.2 in order to see the SA induction and the robust phenotype in response to a high inoculum of bacteria (Figure 2; OD_600_ = 0.2 vs. the inoculum used in Figure 1; OD_600_ = 0.1). Simultaneously, we measured the extent of pathogen growth at the same time point (Figure S2). As a control for the assay, we included the SA biosynthesis mutant *sid2,* which does not accumulate SA in response to pathogens (Wildermuth et al., 2001). High levels of SA accumulated in both *max2* mutants (Figure 2), consistent with our previous measurements of SA after pathogen infection (Piisilä et al., 2015). In contrast, the levels of SA did not increase to significantly higher levels in either strigolactone biosynthesis mutants (*max3*, *max4*) or the receptor (*d14*). At this higher pathogen inoculum and earlier time point (27 hpi vs. 48 hpi), only the *max2* mutants were significantly more sensitive (Figure S2). Therefore, we conclude that the *max2* mutants displayed robust pathogen sensitivity at different infection conditions, while the differences in sensitivity of strigolactone biosynthesis and D14 were only significant with smaller bacterial inoculum at a later time point.

### 3.3 Strigolactone is not a major regulator of defense gene expression

**Figure 3:**
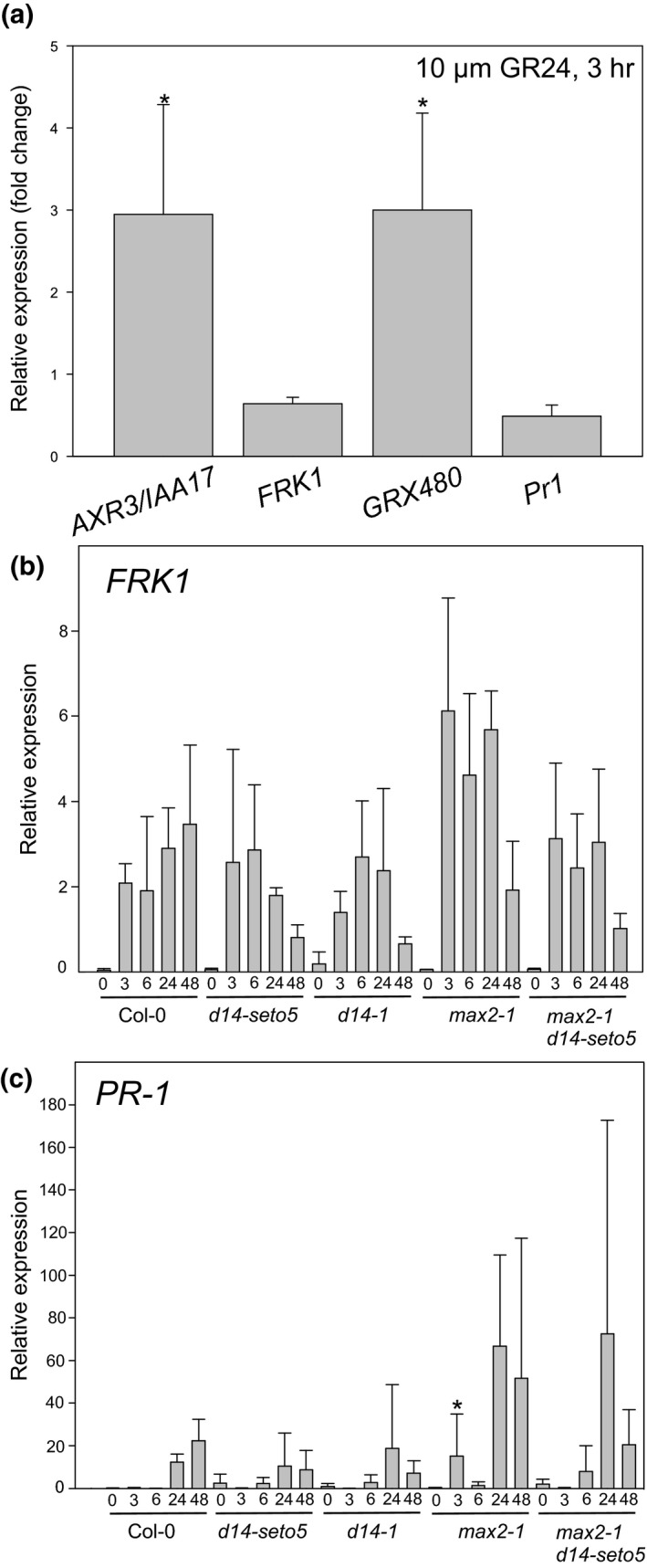
Relative gene expression in response to *Pseudomonas syringae* DC3000 spray infection and GR24 spray (dissolved in DMSO). (a) Relative expression (fold change GR24/control) after 3 hr 10 µM GR24 treatment. The fold change is calculated from three biological repeats. (b‐c) Four‐week‐old plants were spray infected with *P. syringae* DC3000. Relative expression was calculated from three biological replicates for each plant line in each time point. In statistical analysis, we compared if Col‐0 significantly differs from mutants. We conducted a logarithmic transformation on the data and then univariate analysis of variance combined to the Hochberg post hoc test

The role of strigolactones in Arabidopsis is best described in branching (Crawford et al., 2010; Gomez‐Roldan et al., 2008; Goulet & Klee, 2010; Waldie et al., 2014). Microarray analysis to find strigolactone‐regulated genes was previously performed with GR24 (a strigolactone analogue) (Mashiguchi et al., 2009). In that study, 64 genes had significantly altered expression and the magnitude of transcriptomic response (i.e., fold change) in the GR24‐responsive genes was small (Mashiguchi et al., 2009). To test if GR24 can regulate genes related to pathogen defense, we treated 10‐day‐old in vitro grown Col‐0 with 10 µM GR24 for 3 hr and measured gene expression with real‐time quantitative PCR (qPCR). Expression of the well‐established pathogen‐responsive genes *PR1* (*PATHOGENESIS‐RELATED GENE 1*, a late response gene indicating activated SA response, Uknes et al., 1992) and *FRK1* (*FLG22‐INDUCED RECEPTOR‐LIKE KINASE 1*, an early flg22 response marker gene, Asai et al., 2002) decreased, although the differences were not statistically significant. Consistent with the role of strigolactones acting together with auxin in regulation of plant development, expression of *AXR3/IAA17 (AUXIN RESISTANT 3/ INDOLE‐3‐ACETIC ACID INDUCIBLE 17)* increased (Figure 3). Expression of *GRX480* (a glutaredoxin that regulates protein redox state) also increased. Notably, *GRX480* expression is regulated by several stimuli, including SA and ROS (Blanco et al., 2009; Koornneef & Pieterse, 2008; Pieterse, Does, Zamioudis, Leon‐Reyes, & Wees, 2012; Xu, Vaahtera, & Brosche, 2015a).

We also monitored the expression of *PR1* and *FRK1* in *P. syringae*‐infected *max2* and *d14* plants. A few subtle differences were observed including increased expression of PR‐1 at an early time point (3 hpi) in *max2‐1* (Figure 3). As a relatively large variation was observed between different biological repeats, it is possible that some subtle differences between mutants might be obscured, but overall it appears that strigolactone signaling is not a major regulator of defense‐related genes.

### 3.4 Strigolactone perception mutants, but not biosynthesis mutants are ozone sensitive

**Figure 4:**
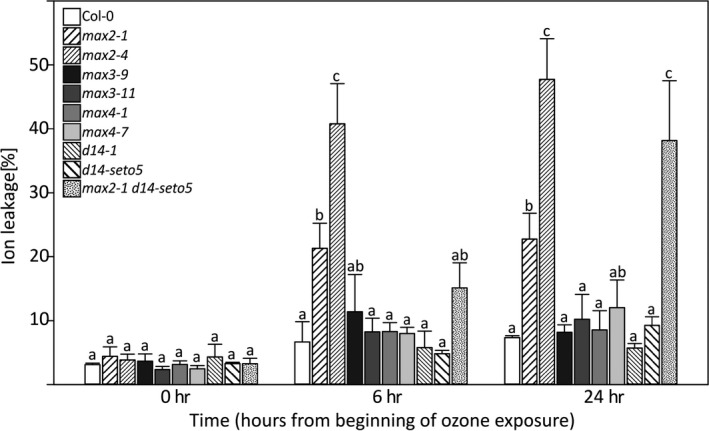
Ozone sensitivity of strigolactone biosynthesis and perception mutants. Ion leakage was measured at indicated time points counted from the beginning of ozone exposure (350 nl/L for 6 hr). In statistical analysis, we conducted a logarithmic transformation on the data and then univariate analysis of variance combined to Hochberg post hoc test. The ion leakage was calculated as percentage of conductance related to the amount of total ions in the sample. The experiment was repeated three times, and there were 4 biological repeats for each plant line in each time point

Treatment of plants with ozone serves as a method to explore plant sensitivity to apoplastic ROS. Ozone enters plants via stomata and immediately degrades to ROS (O_2_^•‐^ and H_2_O_2_) in the apoplastic space, which initiates cell death signaling and leads to development of visible tissue lesions (Overmyer et al., 2018; Vahisalu et al., 2010; Vainonen & Kangasjärvi, 2015). Importantly, as ozone enters the plant through stomata, the mechanisms of sensitivity to this air pollutant can broadly be divided into stomata‐dependent or stomata‐independent mechanisms (Vainonen & Kangasjärvi, 2015; Xu, Vaahtera, Horak, et al., 2015b). Therefore, we assessed the extent of ozone‐induced damage observed after a 6‐hr treatment with 350 nl/L O_3_ by measuring ion leakage before ozone exposure (0 hr) and at two time points (6 hr and 24 hr) after beginning of the treatment (Figure 4). Similarly to phenotypes observed after infection with *P. syringae* (Figure 1a and Figure S2), only the *max2* mutants were ozone sensitive. As also seen in the pathogen infection, the *max2‐4* allele had a stronger phenotype than *max2‐1*. The ion leakage measured 24 hr after beginning of exposure was also higher in *max2‐1 d14‐seto5* double mutant as compared to *max2‐1* or *d14‐seto5* single mutants. The differential response of *max2* versus the biosynthesis and perception mutant suggests that MAX2 might act in additional signaling pathways that extend beyond strigolactone signaling.

### 3.5 Strigolactones do not directly regulate the stomatal aperture

**Figure 5:**
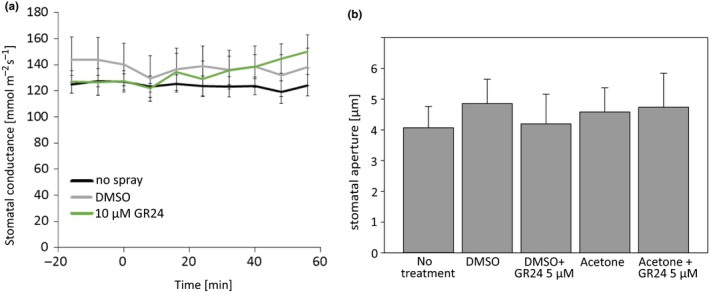
Stomatal response to strigolactone analogue (GR24) spray. (a) Time course of stomatal conductance of WT Col‐0 plants. At time T = 0 min, the plants were sprayed with 10 µM GR24 or mock and returned back to the measuring cuvette. Data are presented as a mean ± *SEM* (*n* = 5). (b) The stomatal aperture width in response to GR24 stock dissolved in DMSO or acetone. The Col‐0 plants were sprayed fully wet with 5 µM GR24 or an equivalent mock solution a day before the stomatal aperture measurements and the differently treated plants were kept covered separately with plastic overnight. Data are presented as a mean ± *SE*. Altogether, approximately 200 stomata were measured from leaves of five sprayed plants, and the experiment was repeated three times with similar results

**Figure 6:**
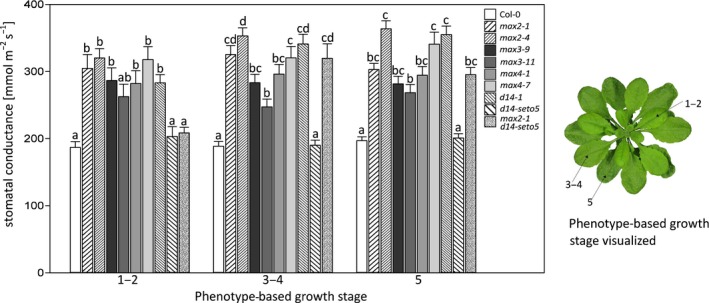
The stomatal conductance of strigolactone biosynthesis and perception mutants measured with a porometer from leaves of different developmental stages. The error bars represent standard error of the mean. 2–3 leaves per each growth stage was measured from each plant, and altogether, a minimum 20 plants were measured from each plant line. The phenotype‐based growth stage is determined in the article by Boyes et al., (2001) in which numbers indicate the growth stage: 1 indicates leaf production, 3 rosette growth, and 5 inflorescence emergence. We used the plants for analysis before they reached the stage 5.10 (i.e., before the first flower buds were visible). In statistical analysis, we conducted a logarithmic transformation on the data and then univariate analysis of variance combined to Tukey HSD post hoc test

**Figure 7:**
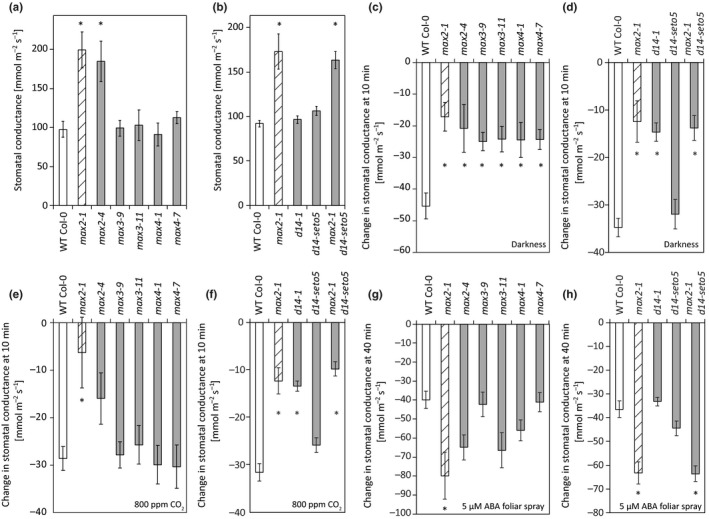
Whole‐plant stomatal conductance and stomatal closure responses to darkness, high CO_2_, and ABA foliar spray. (a) Stomatal conductance of *max2*, *max3,* and *max4* mutants (*n* = 7–11). (b) Stomatal conductance of *max2* and *d14* mutants (*n* = 10–20). (c) Darkness‐induced stomatal closure of *max2*, *max3,* and *max4* mutants (10 min after induction; *n* = 7–11). (d) Darkness‐induced stomatal closure of *max2* and *d14* mutants (10 min after induction; *n* = 10–20). (e) High CO_2_‐induced stomatal closure of *max2*, *max3,* and *max4* mutants (10 min after induction; *n* = 6–12). (f) High CO_2_‐induced stomatal closure of *max2* and *d14* mutants (10 min after induction; *n* = 10–23). (g) ABA‐induced stomatal closure of *max2*, *max3,* and *max4* mutants (40 min after induction; *n* = 6–12). (h) ABA‐induced stomatal closure of *max2* and *d14* mutants (40 min after induction; *n* = 13–23). All graphs present the mean ± *SEM*. Asterisks denote statistically significant differences according to one‐way ANOVA with Tukey HSD post hoc test. The time course data used to calculate the bar graphs (c–h) can be found in Figure S3

**Figure 8:**
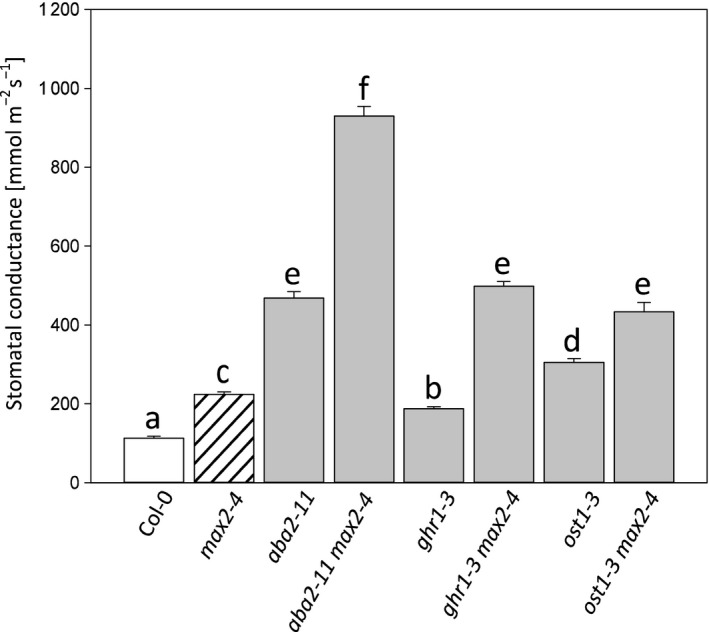
The basal level of whole‐rosette stomatal conductance of double mutants measured with intact plants. Four‐week‐old plants were measured, and the stomatal conductance is the average from 5–6 plants. Data are presented as the mean ± *SEM*. In statistical analysis, we conducted a logarithmic transformation on the data and then univariate analysis of variance combined to Tukey HSD post hoc test

Strigolactone perception and biosynthesis mutants were more sensitive to *Pseudomonas* infection (Figure 1). However, changes in SA accumulation (Figure 2) or gene expression (Figure 3) could not explain this sensitivity. As spray infections require the bacteria to enter through stomata, guard cell signaling leading to stomatal closure may be one of the primary functions of strigolactones. To explore this possibility, we used various stomatal assays and mutants to further study strigolactone‐dependent regulation of guard cell function (Figures 5, 6, 7, 8). First, we tested the ability of GR24 to induce rapid stomatal responses within an hour after application onto leaves. For this, we sprayed the Col‐0 rosettes with 10 µM GR24 (stock dissolved in DMSO and diluted in water) and followed the stomatal conductance by measuring the whole‐rosette stomatal conductance (Kollist et al., 2007; Merilo, Jalakas, Kollist, & Brosché, 2015). However, no change in stomatal conductance after the foliar spray was detected neither in mock‐ nor GR24‐treated plants (Figure 5a). Next, we tested the ability of GR24 to close stomata in a longer time frame. GR24 is not soluble in water; therefore, alternative solvents have been used to dissolve this chemical (DMSO was used for gene expression, Mashiguchi et al., 2009; acetone was used for stomatal aperture measurements, Lv et al., 2018). We prepared a stock of GR24 in DMSO or acetone and diluted to 5 µM in water with 0.02% Silwet‐L77 and sprayed on Col‐0 plants. Stomatal apertures were measured 24 hr after spraying with the use of a protocol formulated by Chitrakar and Melotto (2010). This late time point was selected to identify possible long‐term effects of GR24 on stomatal aperture. However, no significant effect of GR24 on stomatal aperture was observed (Figure 5b). Taken together, our results indicate that treatments with a strigolactone analogue GR24 are not able to induce stomatal closure in intact plants.

### 3.6 Stomatal conductance is higher in strigolactone mutants at different leaf developmental stages

Various types of methods are available to measure stomatal conductance and thus getting an estimate of stomatal aperture. To investigate the stomatal conductance at the developmental stage resolution, we measured stomatal conductance with a porometer, in which the sensor head is clipped onto a single leaf and the conductance is measured from only the abaxial side of the leaf. For this, we used 5‐ to 6‐week‐old plants and measured leaves at different developmental stages 1–2, 3–4, and 5 (Boyes et al., 2001, which roughly corresponds to young, middle age and old, leaves, respectively, Figure 6). Most of the strigolactone perception and biosynthesis mutants had higher stomatal conductance as compared to Col‐0 with no regard to leaf age (Figure 6). The weak *d14‐seto5* did not display higher conductance, and the double mutant *max2‐1 d14‐seto5* showed a differential response with increased conductance only in middle age and older leaves (Figure 6).

### 3.7 Stomatal conductance in intact plants and in response to darkness, high CO_2_, and ABA

Porometer measurements require clipping the sensor head onto the leaf, which could potentially activate stress responses, for example, touch‐induced signaling. To measure the stomatal conductance of intact whole plant rosettes, we used a multi‐cuvette gas exchange system (Kollist et al., 2007), in which the plant is inserted into the machine without any touching. In contrast to results obtained with the porometer (Figure 6), the whole‐rosette stomatal conductivity measurements indicated that only the *max2* alleles (and *max2‐1 d14‐seto5*) displayed increased stomatal conductance (Figure 7a and b).

Next we measured the response to several other stimuli: darkness, high CO_2_, and ABA (Figure 7 and Figure S3) that induce stomatal closure in wild‐type plants (Assmann & Jegla, 2016; Merilo et al., 2013). In response to darkness, both strigolactone biosynthesis (*max3*, *max4*) and perception (*max2*, *d14‐1*) mutants had significantly slower rate of stomatal closure while upon high CO_2_ treatment the response was impaired in *max2* and *d14* mutants (Figure 7e and f). In contrast, the stomatal response to ABA was not impaired in any of the mutants, instead, an enhanced stomatal closure rate was observed in *max2‐1*, possibly due to higher stomatal conductance of *max2‐1* before ABA treatment (Figure 7g and h).

Most of our knowledge on regulation of guard cell signaling is in the context of ABA signaling (Merilo et al., 2018), and very few regulators specific for other stimuli have been found (Engineer et al., 2016). Thus, the differential response of especially *max2* with a defective response toward high CO_2_ and darkness, and normal to enhanced response toward ABA, adds a new regulatory layer in guard cell signaling. As an F‐Box protein, MAX2 might be involved in the targeted degradation of some other regulatory component in guard cell signaling.

### 3.8 MAX2 regulates stomatal function independently of ABA signaling

Multiple results obtained in this study indicated significant differences between *max2* and other strigolactone signaling‐related mutants, that is, the *max2* mutant was the only mutant exhibiting ozone sensitivity and consistently higher stomatal conductance (Figures 4, 6 and 7). To further explore the function of MAX2 in the guard cell signaling network, we crossed *max2* with mutants defective in ABA biosynthesis (*aba2*), guard cell ABA signaling (*ost1*), and a scaffold protein GHR1 (*ghr1*) that is required for activation of the guard cell anion channel SLAC1. Next, single and double mutants were subjected to measurement of stomatal conductance. Interestingly, all double mutants had significantly higher stomatal conductance than the corresponding single mutants (Figure 8), suggesting that MAX2 acts in a signal pathway that functions in parallel to the well‐characterized stomatal ABA signaling pathway.

## 4 DISCUSSION

Plant defense responses to pathogen infection are highly complex and include many different signaling pathways (Koornneef & Pieterse, 2008; Pieterse et al., 2012). While hormones associated with stress, for example, salicylic acid, jasmonic acid, ethylene, and abscisic acid have long been studied for their role in pathogen responses, hormones typically associated with development influence the outcome of plant–pathogen interactions (Pieterse, Leon‐Reyes, Ent, & Wees, 2009). While the function of strigolactones in Arabidopsis was initially characterized for their role in shoot branching (Bennett et al., 2006, Hayward et al., 2009, Stirnberg, Furner, & Leyser, 2007), they appear to be important in pathogen sensitivity. Mutants involved in strigolactone sensing (*max2*, *d14*) and biosynthesis (*max3* and *4*) were more sensitive to *P. syringae* DC3000 spray infection than Col‐0 (Figure 1b). Pathogen infection assays can be performed in many different ways, with different levels of inoculums and different delivery methods to the plants (e.g., spray infection or syringe infiltration). We designed our experiment to use different levels of inoculum to test the reproducibility of the observed pathogen sensitivity phenotype. This revealed a differential response, where only *max2* showed significantly increased sensitivity at higher inoculum (Figure S2). This suggests that the role strigolactone in pathogen responses is condition‐dependent and its function is better described as a modulator of the defense response rather than a master regulator. Similarly, the very modest transcriptional response of pathogen defense genes to the application of the synthetic strigolactone analogue GR24 (Figure 3 and Mashiguchi et al., 2009) also indicates that strigolactones are not direct regulators of defense signaling. At the same time, as the *max2* phenotype is very robust, this suggests a broader role for MAX2 in several signaling pathways in addition to strigolactone signaling. Moreover, the high levels of SA that accumulated in both *max2* alleles in response *P. syringae* DC3000 spray (Figure 2) were consistent with the results in Piisilä et al. (2015) where SA levels were measured with gas chromatography combined with mass spectrometry.

**Figure 9:**
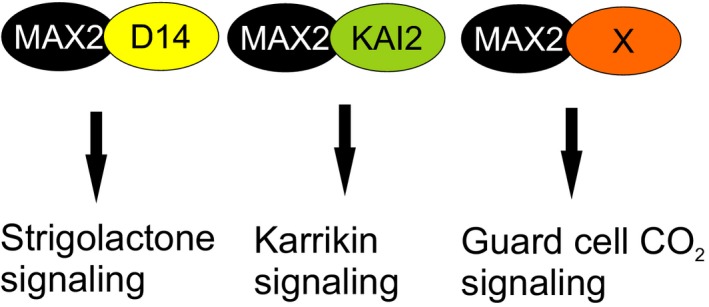
MAX2 acts in different signaling pathways to target proteins to ubiquitin‐mediated degradation. In strigolactone signaling, MAX2 interacts with the strigolactone receptor D14 (Seto et al., 2019). In karrikin signaling, MAX2 interacts with the karrikin receptor KAI2 (Guo, Zheng, Clair, Chory, & Noel, 2013; Li et al., 2017). In guard cell signaling, a proposed new function for MAX2 is to target a component X in guard cell CO_2_ signaling to degradation

We propose that the role of strigolactone signaling components, especially MAX2, in plant defense, is related to the regulation of stomatal function, which subsequently influences pathogen and defense responses. This was clearly indicated by an increased ozone sensitivity of *max2* mutant plants (Figure 4). Arabidopsis mutants and accessions with more open stomata are ozone sensitive as more ozone can enter the plant (Brosché et al., 2010; Overmyer et al., 2008). Similarly, more open stomata would also allow higher entry of pathogenic bacteria, for example, *P. syringae*. Additionally, the impaired stomatal closure in response to *P. syringae* infection likely contributes to sensitivity (Piisilä et al., 2015). In pathogen and ozone sensitivity studies, the strong *max2‐4* allele was clearly more impaired than the weak *max2‐1*. When weak alleles of *max2* (*max2‐1*) and *d14* (*d14‐seto5*) were combined in the *max2‐1 d14‐seto5* double mutant, a similar phenotype to the strong *max2‐4* was observed in pathogen (48 hpi) and ozone (24 hr) responses (Figures 1 and 3). This is consistent with the current understanding of D14 and MAX2 interacting in strigolactone perception (Lv et al., 2018). However, the fact that *d14‐1* and strigolactone biosynthesis mutants *max3* and *max4* were not ozone sensitive suggests that MAX2 has a more versatile role in stress responses than the other strigolactone signaling‐related proteins. Previously, MAX2 was also shown to have a role in karrikin signaling (Li et al., 2017), pointing toward different signaling roles for this F‐box protein, possibly by targeting the degradation of proteins in different signaling pathways (Figure 9).

To further evaluate the role of strigolactones in regulation of guard cell signaling, we used several different methods and genetic tools. To our surprise, we could not observe GR24‐induced stomatal closure in either stomatal aperture or stomatal conductance assays (Figure 5). Ha et al. (2014) rescued the drought phenotype of the strigolactone biosynthesis (*max3* and *max4*) mutants with strigolactone spray. Moreover, by using epidermal peels, Lv et al. (2018) showed that the stomata close in response to GR24 and strigolactones were concluded to be common regulators of stomatal closure *in planta* (Zhang, Lv, & Wang, 2018). One challenge in using GR24 is that no standard method for dissolving this chemical is established, and possibly, the solvent of GR24 might affect the results. Thus, we dissolved GR24 both in DMSO and acetone but neither of the solutions resulted in clear differences from mock in the size of stomatal aperture or in stomatal conductance.

There are several methods to measure stomatal function. First, we performed a classical porometer measurement in order to explore if the growth stage (or age) of the leaves affected stomatal conductance. All of the strigolactone biosynthesis and perception mutants had a higher stomatal conductance except for *d14‐seto5* (Figure 6) that consistently had weaker phenotypes than the other mutants, which is also evidenced by its growth phenotype (Figure S4). As the strigolactone mutants have a “bushy” phenotype (Figure S4), with leaves often laying on top of each other, the porometer measurements have the advantage of measuring a specific area independent from the rest of the plant. However, the data provided by the porometer are also limited, since the porometer sensor area is rather small, and thus, only a small area of a leaf is measured and we only measured the abaxial side of the leaf.

To complement the porometer data, we used a custom‐made device that measures the whole‐rosette gas exchange and allows parallel analysis of 8 intact plants and their real‐time responses to stomata‐affecting factors, such as CO_2_ concentration, darkness, and the phytohormone ABA (Kollist et al., 2007). In contrast to porometer measurements, only *max2* and *max2 d14* mutants had increased stomatal conductance when the intact whole rosettes were measured (Figure 7a and b). These lines displayed also increased ozone‐induced cell death confirming earlier reports where increased stomatal conductance has led to increased ozone sensitivity of different Arabidopsis accessions and mutants (Brosché et al., 2010; Overmyer et al., 2008). The difference in results obtained with these two methods might have several explanations: (a) in promoter measurements, only a small leaf area is measured, and thus, there is large edge‐to‐area ratio which can be source of error (Long & Bernacchi, 2003), besides Arabidopsis leaves are vulnerable and clamping them to measuring head could lead to wounding. (b) There are pitfalls in whole‐rosette gas exchange measurements as well. There is always significant variation of leaf ages, and some extent shading between neighboring leaves can occur. These constraints can create a microclimate and variation in leaf temperatures, which is a key input for calculation of stomatal conductance. Accordingly, it is not correct to compare numerical values of whole‐plant gas exchange with those of leaf porometry. The challenges of accurate gas exchange measurements are further discussed in Long, Farage, & Garcia, 1996 and Long & Bernacchi, 2003. Transpiration is another broadly used physiological parameter to estimate plant water transport, and calculation of transpiration is more robust as it does not require values for leaf temperature. We also calculated whole‐plant transpiration of the studied mutants, and this analysis led to the same result, that is, transpiration was significantly higher only in *max2* and *max2 d14* lines (Figure S5)*.* (c) As porometer measurements were performed at the University of Helsinki, and the whole‐rosette assays at the University of Tartu, other factors affecting growth conditions could sensitize the biosynthesis mutants (*max3*, *max4*) to have more open stomata in the Helsinki growth conditions. Further research might resolve this issue, but given the consistent phenotype of *max2* across several different assays and growth conditions, the response of this mutant strongly suggests an important role for MAX2 in guard cell signaling. Increased stomatal conductance can result from either increased stomatal aperture or stomatal density. In *max2‐1* and *max2‐4*, we previously observed increased stomatal aperture compared to wild type (Piisilä et al., 2015). However, given the different results in stomatal conductance, stomatal aperture and responses observed for the strigolactone‐related mutants between different methods and growth conditions (Figures 6 and 7; Bu et al., 2014; Ha et al., 2014; Lv et al., 2018), we cannot exclude that the increased stomatal conductance could be a result of both increased aperture as well as increased number of stomata.

Testing stomatal responses to several different treatments showed that high CO_2_‐induced stomatal closure was impaired in *max2 *and* d14‐1* (Figure 7e and f). A sudden darkness treatment during the normal light period is partially initiated by the same mechanism as CO_2_ signaling. Removal of light stops photosynthesis and this leads to increase of CO_2_ concentration inside the leaf, similar to the situation when elevated CO_2_ is applied. The darkness‐induced stomatal closure was reduced in *max2, max3, max4,* and *d14‐1*; that is, the response to darkness was more broadly impaired than the response to high CO_2_. Of the different stimuli and treatments that lead to stomatal closure, the response to darkness might be the least studied. Thus, the impaired darkness response in both strigolactone biosynthesis and perception mutants opens the possibility for further studies into this branch of guard cell signaling.

To further study the relationship between strigolactone and ABA, we examined whether ABA signaling and *MAX2* share the same elements in guard cell signaling. For this, we crossed *max2* with other guard cell signaling mutants *ost1*, *ghr1,* and the ABA biosynthesis mutant *aba2.* The resulting double mutants (*max2 ghr1*, *max2 ost1,* and *max2 aba2*) had a higher stomatal conductance than any of these mutants individually. Thus, it appears that MAX2 functions on a pathway that is parallel to ABA signaling (see also Lv et al., 2018). As very few regulators of ABA‐independent guard cell signaling have been found (Assmann & Jegla 2016, Engineer et al., 2016), the impaired CO_2_ and darkness response in *max2* implicate that the F‐Box protein MAX2 has a crucial role in targeting as yet unidentified important guard cell regulator to ubiquitin‐mediated protein degradation (Figure 9). This regulator would not be any of the well‐known components of the ABA signaling pathway, for example, the PYR/PYL receptors, PP2C phosphatases, or OST1 kinase (Assmann & Jegla 2016, Engineer et al., 2016). A future screen for MAX2‐interacting proteins using, for example, MAX2 co‐immunoprecipitation from isolated guard cells could be used to unravel other components of this specific branch of guard cell signaling and give new information on how different signaling pathways interact to regulate stomatal function.

## CONFLICT OF INTEREST

The authors declare no conflict of interest associated with the work described in this manuscript.

## AUTHOR CONTRIBUTIONS

M.K., L.J., V.P., E.T.P., T.K., H.K., and M.B. designed experiments. M.K. and M.B. wrote the manuscript. M.K., L.J., V.P., C.W., H.K., and M.B edited the manuscript. M.K., P.D., and V.P performed pathogen assays. M.K. performed ozone assays. M.K. and P.D. performed porometer analysis. M.K. and M.B. performed qPCR experiments. M.K., L.J., D.Y., and O.Z. performed gas exchange analysis. C.W. generated double mutants. M.K., L.J., V.P., C.W., H.K., and M.B analyzed the data.
