# Supplementary figures and images for: Differential role of MAX2 and strigolactones in pathogen, ozone, and stomatal responses
Source: Plant Direct. 2020 Feb 28;4(2):e00206. doi: 10.1002/pld3.206 (PMC7047155; doi:10.1002/pld3.206)

## Supporting Information Figure 1

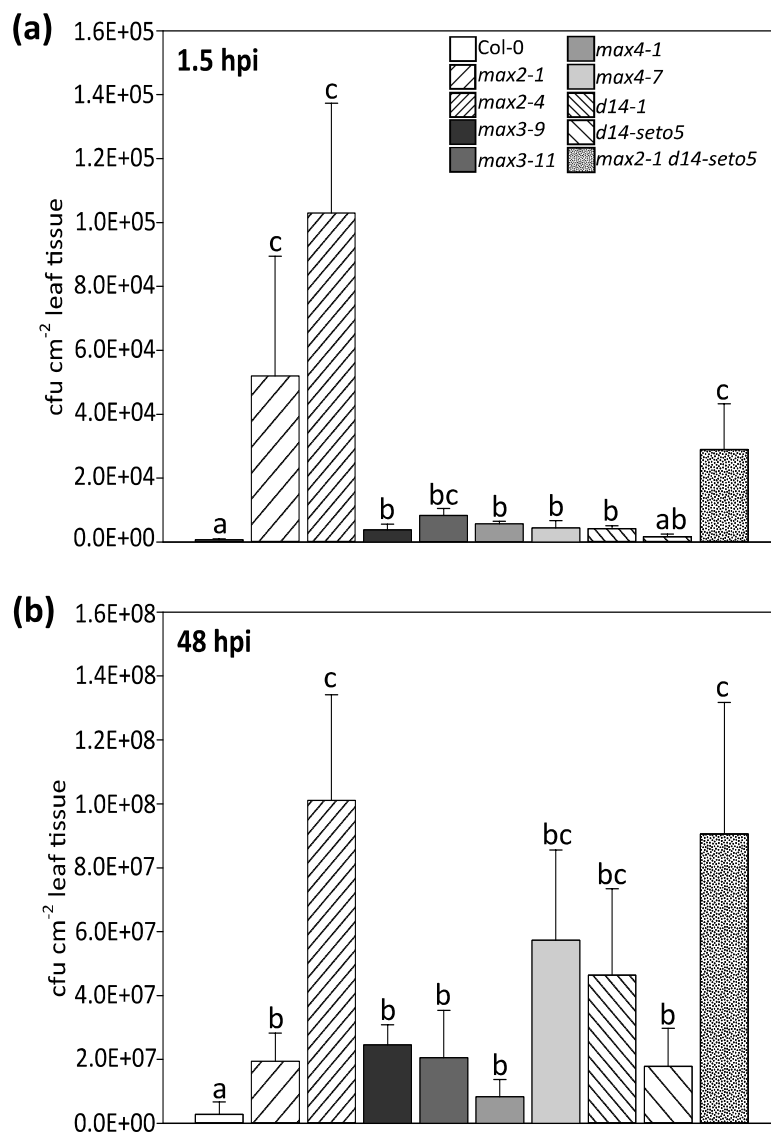

Supplement: Supplementary file 1 [file PLD3-4-e00206-s001.pdf]

Supporting Information Figure 2

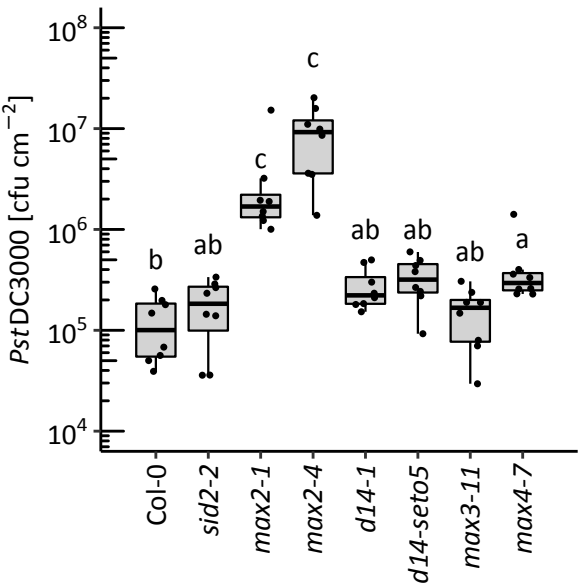

Supplement: Supplementary file 2 [file PLD3-4-e00206-s002.pdf]

Supporting Information Figure 3

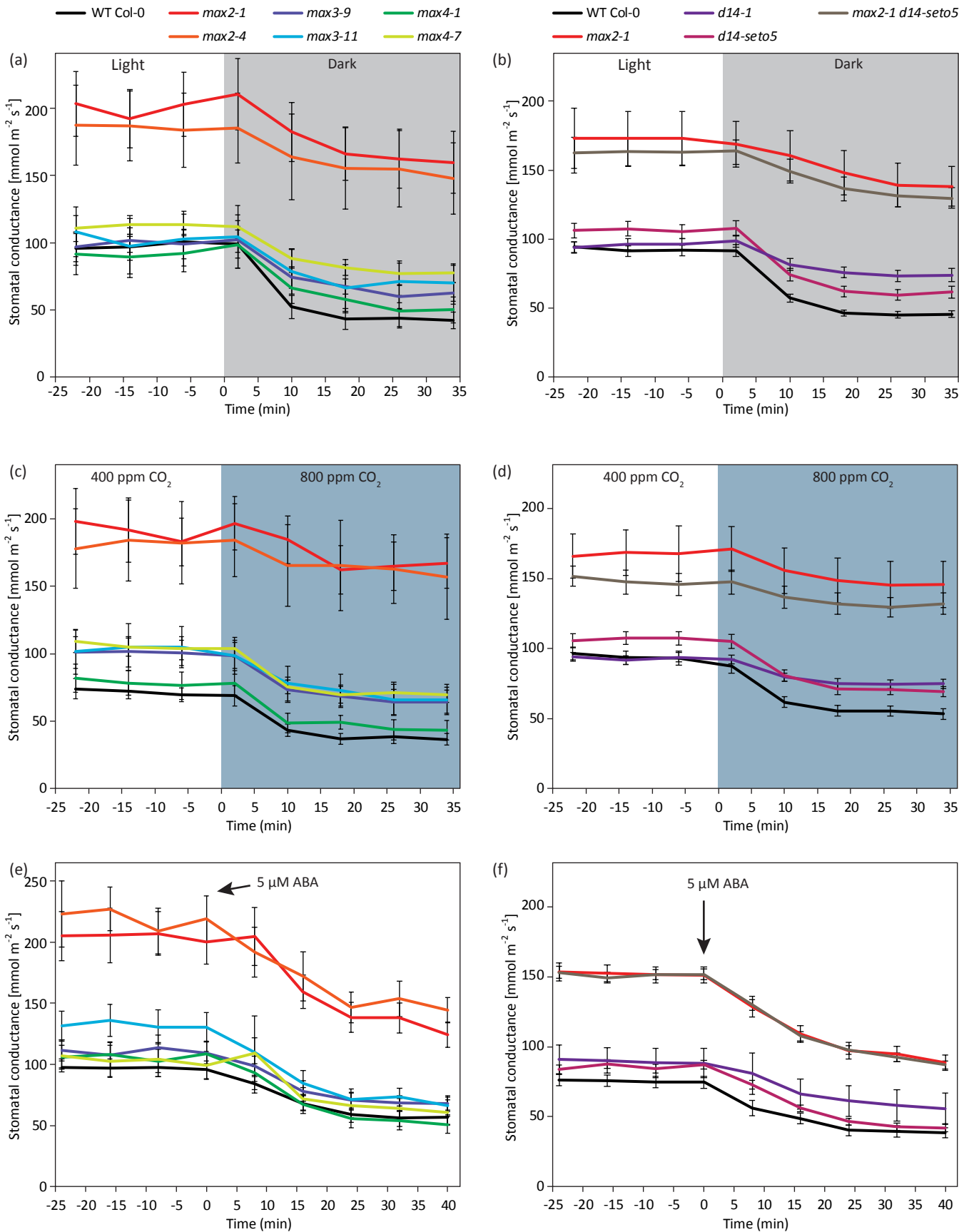

Supplement: Supplementary file 3 [file PLD3-4-e00206-s003.pdf]

Supporting Information Figure 4

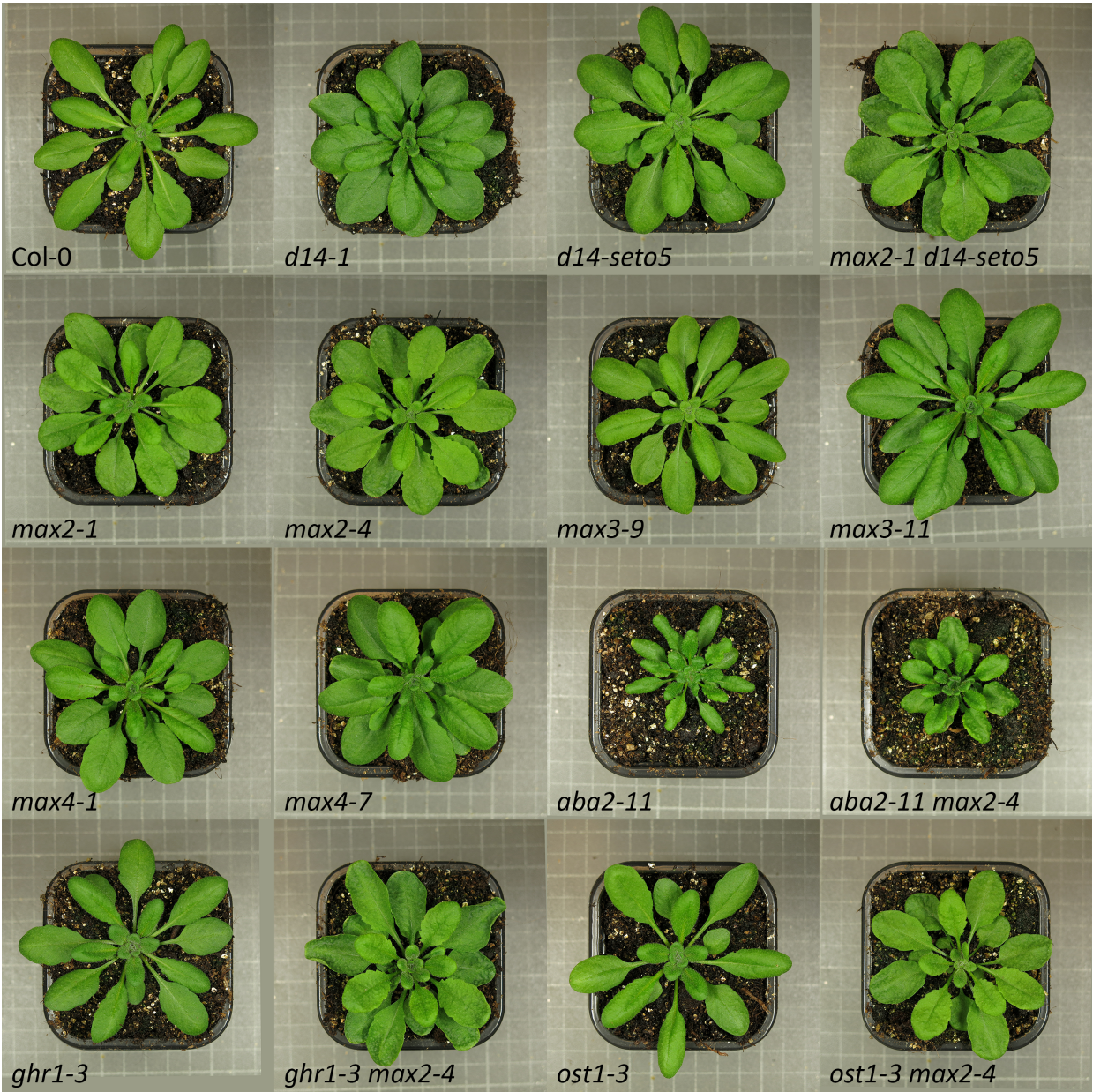

Supplement: Supplementary file 4 [file PLD3-4-e00206-s004.pdf]

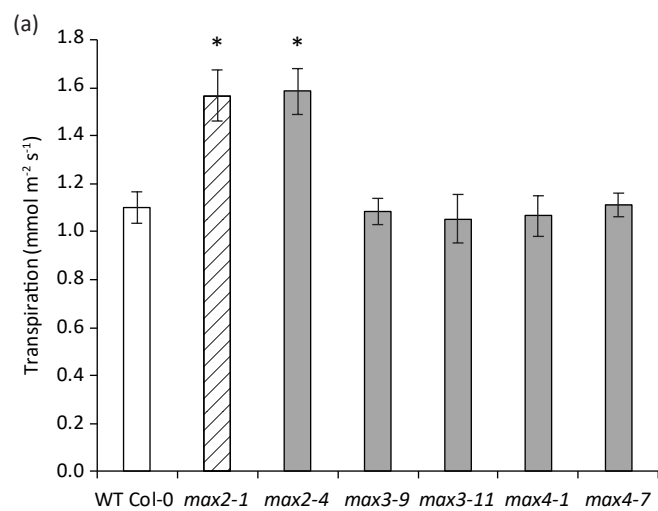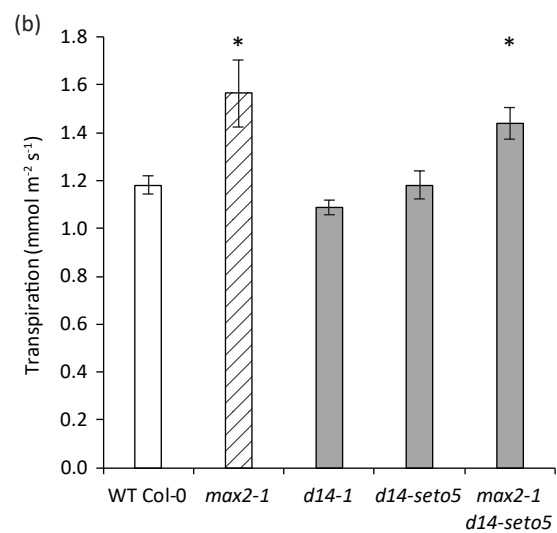

Supplement: Supplementary file 5 [file PLD3-4-e00206-s005.pdf]
