# Supplementary material for: Differential role of MAX2 and strigolactones in pathogen, ozone, and stomatal responses
Source: Plant Direct. 2020 Feb 28;4(2):e00206. doi: 10.1002/pld3.206 (PMC7047155; doi:10.1002/pld3.206)
Supplement: Supplementary file 6 [file PLD3-4-e00206-s006.pdf]

Supplemental Table 1. Primers used in this study

Primer name and description	Primer Sequence (5' → 3')	Comments
**Genotyping of mutant lines**		
D14-seto5 F	TATGATCGGAATCATCGCTTC	
D14-seto5 R	GACCTAAGATACTCCGCCACC	
D14-1 MW221	AAGAATATGGCAAGTGCAAC	Genomic primer for Atd14-1 allele, Waters et al 2012
D14-1 MW222	GATGATTCCGATCATAGCG	Genomic primer for Atd14-1 allele, Waters et al 2012
L4_WiscLoxHS	TGATCCATGTAGATTTCCCGGACATGAAG	T-DNA primer for Atd14-1 allele, Waters et al 2012
max2-1 F	GTTGGAGAATTTTTGACGCCAT	
max2-1 R	CAAAGGCCGAGCTGTAAAGAGA	
max2-4 F	TACCTCCTGTGCTTCTCCGCTA	SALK_028336
max2-4 R	CTTCTGGCTCCTCTCGTAACCA	SALK_028336
max3-9 F	CCCTTCCGGTACATACTACTT	
max3-9 R	GTAGTGAGTATCCGTGAATGC	
max3-11 F	CGTTAAACCCAAGCAACGAGAG	SALK_023975
max3-11 R	CATCGGAAACGTCAAATTCCTC	SALK_023975
max4-1 F	AAGGAAAGATACCCACTTGGC	
max4-1 R	GTACCAACCATGCCTATGACC	
SPM8225F	TCGGCTTATTTCAGTAAGAGTG	SPM8225F transposon insertion primer for max4-1, Sorefan et al 2003
max4-7 F	ACTGGTCATAGGCATGGTTGGT	SALK_082552
max4-7 R	ACCGAATATCTCAGGGGCATTT	SALK_082552
aba2-11_F	TTGATGTGACAGGCTTTTGG	
aba2-11_R	TCTTGCAGATCAACAATGCAG	
ghr1-3_F	TGGCCCTTCTAAGCTGTTAGA	
ghr1-3_R	CTCCTTATCTGGACCTTTGCC	
ost1-3_F	CATATCTTTAGACGAGGGGCC	
ost1-3_R	GTGAGTGGTCCAATGGATTTG	
SALK_LBb1.3	ATTTTGCCGATTTCGGAAC	
GK_o8474	ATAATAACGCTGCGGACATCTACATTTT	
**qPCR primers**		
YLS8 for	TTACTGTTTCGGTTGTTCTCCATTT	efficiency 1.822
YLS8 rev	CACTGAATCATGTTCGAAGCAAGT	
PP2AA3 for	GCGGTTGTGGAGAACATGATACG	efficiency 1.854
PP2AA3 rev	GAACCAAACACAATTCGTTGCTG	
TIP41 for	GTGAAAACTGTTGGAGAGAAGCAA	efficiency 1.867
TIP41 rev	TCAACTGGATACCCTTTCGCA	
AXR3-F	CACATTGATTGGTGTTAATTGC	efficiency 1.744
AXR3-R	CCAAATCCAGATCAAAACACAG	
GRX480 for	ACGGAGAGGATGTTGCATGTGTC	efficiency 1.843
GRX480 rev	AATCTCAAGGACCGCCGGATTC	
PR-1 for	CGGAGCTACGCAGAACAACT	efficiency 1.658
PR-1 rev	CTCGCTAACCCACATGTTCA	
NCED3 for	AGCTCCTTACCTATGGCCAGT	efficiency 1.968
NCED3 rev	CGCTCTCTGGAACAAATTCATC	
FRK1 for1	CGGTCAGATTTCAACAGTTGTC	efficiency 1.843, used in GR24 assay (Figure 3a)
FRK1 rev1	AATAGCAGGTTGGCCTGTAATC	
FRK1 for2	GCCAACGGAGACATTAGAG	efficiency 1.876, used in P.syringae infection experiment (Figure 3b), primer sequences published by Yeh et al., (2015)
FRK1 rev2	CCATAACGACCTGACTCATC	

References

Yeh Y.H., Chang Y.H., Huang P.Y., Huang J.B., Zimmerli L. 2015. Enhanced Arabidopsis pattern-triggered immunity by overexpression of cysteine-rich receptor-like kinases. Front. Plant Sci., 6:322.
